# Ischemic preconditioning promotes hepatic differentiation in human liver organoids

**DOI:** 10.3389/fcell.2026.1862631

**Published:** 2026-07-03

**Authors:** Maura Cimino, Rosaria Tinnirello, Andrea Orlando, Simone Messina, Salvatore Gruttadauria, Duilio Pagano, Pier Giulio Conaldi, Alessandro Mattina, Riccardo Perriera, Giovanni Zito, Massimo Pinzani, Vitale Miceli

**Affiliations:** 1 Department of Research, IRCCS ISMETT (Istituto Mediterraneo per i Trapianti e Terapie ad alta specializzazione), Palermo, Italy; 2 Department of Biomedicine, Neurosciences and Advanced Diagnostic (BiND), University of Palermo, Palermo, Italy; 3 Abdominal Center, IRCCS ISMETT (Istituto Mediterraneo per i Trapianti e Terapie ad alta specializzazione), Palermo, Italy; 4 UPMC Italy, Palermo, Italy; 5 Department of General Surgery and Medical-Surgical Specialties, University of Catania, Catania, Italy; 6 Diabetes Service, IRCCS ISMETT (Istituto Mediterraneo per i Trapianti e Terapie ad alta specializzazione), Palermo, Italy

**Keywords:** hepatocyte differentiation, human liver organoids, ischemia-reperfusion injury, liver regeneration, liver transplantation

## Abstract

**Introduction:**

Liver regeneration is essential for successful outcomes after liver transplantation. However, ischemia-reperfusion injury (IRI) remains a major determinant of graft dysfunction that can profoundly affect hepatic regenerative responses. Although ischemic stress has been implicated in both tissue damage and regenerative signaling, its direct impact on progenitor differentiation and hepatocyte maturation remains poorly understood, partly due to the lack of controllable human experimental models. In this study, we investigated how controlled ischemic stress influences hepatocyte differentiation and regenerative signaling using a human liver organoid (HLiO) model.

**Methods:**

Organoids were expanded as undifferentiated cultures and subjected to a stepwise differentiation protocol toward hepatocyte-like cells. An *in vitro* IRI model was generated by exposing organoids to 16 h of cold ischemia (O_2_ 0%) followed by reperfusion under normoxic conditions (O_2_ 20%). Molecular, imaging, and functional analyses were performed to evaluate progenitor status, hepatocyte differentiation, and the release of inflammatory mediators.

**Results:**

Differentiation of HLiOs induced a shift from progenitor-associated gene expression toward hepatocyte-specific programs, accompanied by increased albumin secretion and expression of mature markers. Controlled ischemia caused a transient reduction in viability and triggered the release of High Mobility Group Box 1 (HMGB1), Interleukin 1 Beta (IL-1β), Interleukin 8 (IL-8), and Oxidized Low Density Lipoprotein Receptor 1 (LOX-1), followed by recovery during reperfusion. Notably, ischemic preconditioning enhanced hepatocyte maturation, characterized by stronger downregulation of progenitor markers, increased expression of Cytochrome P450 3A4 (CYP3A4), Hepatocyte Nuclear Factor 4 Alpha (HNF4A), Alpha-1 Antitrypsin (A1AT), and albumin, and improved functional output compared with standard differentiation.

**Discussion:**

These findings suggest that sub-lethal ischemic stress may act as a regenerative stimulus, potentially mediated by a progenitor-associated HMGB1–LOX-1–IL-8 signaling axis. Despite the absence of non-parenchymal liver cells, this organoid platform provides a controllable system to study intrinsic regenerative responses to ischemia, and indicates that appropriately modulated ischemic cues might promote hepatocyte differentiation, and improve graft recovery after liver transplantation.

## 1 Introduction

Liver transplantation (LTx) is the only life-saving therapy for patients with end-stage liver disease, acute liver failure, or selected hepatic malignancies (European Association for the Study of the, Liver (EASL), 2024). Although advances in surgical techniques and perioperative management have improved outcomes (European Association for the Study of the Liver (EASL), 2024; Ashwat et al., 2025), ischemia-reperfusion injury (IRI) remains a major determinant of graft dysfunction and long-term transplant success (Liu and Man, 2023). This issue is particularly relevant with the increasing use of extended-criteria donor grafts, which are more susceptible to ischemic damage (Finotti et al., 2024; Xiang et al., 2024; Satish et al., 2025; Colmenero et al., 2026). During organ procurement and reperfusion, ischemic stress triggers metabolic disruption, oxidative injury, and inflammatory responses that negatively affect graft quality (Zhao et al., 2025; Calligaris et al., 2026). In this context, dynamic machine perfusion strategies have emerged as promising procedures to mitigate IRI (Gruttadauria et al., 2025; Patrono et al., 2025). Moreover, while several pharmacological approaches have demonstrated encouraging protective effects in preclinical models of hepatic IRI, including anti-apoptotic agents and other experimental strategies (Zhang et al., 2024b), their clinical translation has remained limited, and effective targeted therapies are still lacking.

Beyond causing tissue damage, IRI strongly influences liver regeneration, a process essential for graft recovery after transplantation (Gomez et al., 2007; Maspero et al., 2023; Huwyler et al., 2025). While excessive or prolonged IRI impairs regenerative responses through hepatocyte injury and sustained inflammatory signaling (Liu and Man, 2023), increasing evidence suggests that controlled ischemic stress may also activate adaptive pathways involved in tissue repair and regeneration (Gomez et al., 2007; He et al., 2009; Maspero et al., 2023; Nwaduru et al., 2025). However, the mechanisms through which ischemia regulates progenitor activation and hepatocyte maturation remain poorly understood, particularly whether ischemic stimuli promote maintenance of a progenitor-like state or accelerate terminal differentiation. Addressing these questions *in vivo* remains challenging because of the complexity of systemic, immune, and hemodynamic influences. To overcome these limitations, physiologically relevant human *in vitro* models are required.

Human liver organoids (HLiOs) have recently emerged as powerful tools for the study of liver development, regeneration, and disease (Lo Nigro et al., 2021; Afonso et al., 2024). Derived from adult or pluripotent stem cells, these three-dimensional structures recapitulate the key features of the human liver, including progenitor cell hierarchies, differentiation capacity, and functional maturation. Liver organoids offer a controllable *ex vivo* platform to dissect cell-intrinsic responses to defined stimuli, such as ischemic stress, independent of systemic influences.

In this study, we established an *ex vivo* model of liver regeneration using undifferentiated HLiOs to investigate the effect of ischemic injury on the hepatic regenerative potential. By exposing organoids to controlled cold ischemia prior to differentiation, we aimed to assess whether ischemic stress influences the balance between progenitor activation and terminal hepatocyte maturation. Through comprehensive morphological, molecular, and functional analyses we sought to determine whether ischemia, when appropriately managed, may not only impair but also modulate regenerative potential offering therapeutic benefits. This approach provides novel insights into the interplay between ischemia and liver regeneration, and supports the use of human liver organoids as a relevant platform to explore strategies aimed at improving graft recovery and outcomes in LTx.

## 2 Materials and methods

### 2.1 Human liver organoid generation and culture

Liver biopsies were obtained during tumor resection or liver transplantation with informed consent in accordance with the Declaration of Helsinki. Written informed consent and details of the procedure were approved by the Institutional Research Review Board of the IRCCS ISMETT (project identification code: IRRB/13/17). Biopsies were minced and digested in EBSS (Thermo Fisher Scientific, United States) containing collagenase D (2.5 mg/mL) and DNase I (0.1 mg/mL) (Roche, Germany) for 20–40 min at 37 °C. Digestion was stopped with cold DMEM (supplemented with Fetal Bovine Serum, FBS 10%) and the suspension filtered through a 70-µm strainer (BD Falcon, United States). Cells were labeled for CD326 (EpCAM) and sorted by FACS (FACSAria, BD Biosciences, United States). EpCAM^+^ cells were centrifuged at 300 *g* for 5 min, and washed with cold advanced DMEM/F12 (AdDMEM/F12) (Thermo Fisher Scientific, United States). The isolation and characterization of the EpCAM^+^ progenitor-enriched population used for HLiO generation has been previously described and validated in our earlier work (Lo Nigro et al., 2021), and the same protocol was applied in the present study. The cell pellet was mixed with Cultrex Basement Membrane Extract, Type 2, (BME 2) (Bio-Techne, United States), and 6,000–12,000 cells were seeded per well in a 24-well plate (BD Falcon, United States). After BME two solidified at 37 °C for 15 min, culture medium (expansion medium, EM) for organoid expansion was added. This medium was composed of AdDMEM/F12 supplemented with 1% glutamax, 10 mM HEPES, 1% of both N2 and B27 supplements (without vitamin A), 1.25 mM N-Acetylcysteine, 10 mM Nicotinamide, 10 nM gastrin, 50 ng/mL EGF, 100 ng/mL FGF10, 25 ng/mL HGF, 25 ng/mL Noggin, 500 ng/mL RSPO1, 5 µM A83-01, 0.5 µM CHIR99021, 10 µM Forskolin, and 10 µM Y27632 (Thermo Fisher Scientific, United States). Undifferentiated HLiOs were cultured for 10–14 days, mechanically and enzymatically dissociated using TrypLE (Thermo Fisher Scientific, United States) into a single cell suspension, and split (1:4–1:8) in fresh BME two every 7–10 days.

### 2.2 Human liver organoid differentiation

Liver organoids were cultured for 5 days in expansion medium (EM) supplemented with BMP7 25 ng/mL (Thermo Fisher Scientific, United States). The medium was then changed into the differentiation medium (DM), which consisted of AdDMEM/F12 supplemented with 1% of both N2 and B27, 50 ng/mL EGF, 10 nM gastrin (Sigma-Aldrich, United States), 25 ng/mL HGF, 100 ng/mL FGF19, 500 nM A83-01, 10 µM DAPT, 25 ng/mL BMP7, and 30 µM dexamethasone (Thermo Fisher Scientific, United States). The differentiation medium was changed every 2–3 days for 13–15 days of culture. Functional analysis was performed on the collected supernatant or whole organoids.

### 2.3 Model of cold ischemia and reperfusion injury

To mimic IRI *in vitro*, HLiOs were subjected to a controlled cold ischemia and reperfusion protocol. Briefly, the ischemic phase was induced by incubating HLiOs in a preservation solution Servator-B (S.A.L.F. S.p.A. Pharmacological Laboratory, Italy), a University of Wisconsin (UW)-equivalent preservation solution, at 4 °C for 16 h under anoxic conditions (0% O_2_ and 0% CO_2_). The duration of cold ischemia was tested over a range of 12–24 h. An ischemic period of 16 h was selected as optimal, as it resulted in approximately 50% reduction in cell viability, followed by complete recovery after 24 h of reperfusion. Following ischemia, to simulate controlled oxygenated rewarming, organoids were pre-warmed at room temperature (20 °C–25 °C) for 30 min, after which reperfusion was simulated by replacing the preservation solution with complete EM, and culturing the organoids at 37 °C for 24 h under normoxic conditions (20% O_2_ and 5% CO_2_). Bright-field images of the HLiOs were acquired using the EVOS M5000 imaging system (Thermo Fisher Scientific, United States). Analyses were performed at predefined experimental endpoints established from previously validated culture and differentiation protocols, rather than by daily longitudinal monitoring.

### 2.4 Gene expression analysis

Real-time quantitative PCR (RT-qPCR) was performed to assess mRNA expression, using SYBR Select Master Mix, according to the manufacturer’s instructions (Thermo Fisher Scientific, United States). A panel of genes associated with the liver progenitor cell phenotype and the mature hepatocyte phenotype was analyzed. Liver progenitor cell markers included: LGR5 (forward primer: GAG​GAT​CTG​GTG​AGC​CTG​AGA​A; reverse primer: CAT​AAG​TGA​TGC​TGG​AGC​TGG​TAA; GenBank: NM_003667.4); EpCAM (forward primer: CTG​GCC​GTA​AAC​TGC​TTT​GT; reverse primer: AGC​CCA​TCA​TTG​TTC​TGG​AG; GenBank: NM_002354.3); A1AT (forward primer: AGG​GCC​TGA​AGC​TAG​TGG​AT; reverse primer: TCC​TCG​GTG​TCC​TTG​ACT​TC; GenBank: NM_001127702.2); CYP3A4 (forward primer: TTC​CTC​CCT​GAA​AGA​TTC​AGC; reverse primer: GTT​GAA​GAA​GTC​CTC​CTA​AGC​T; GenBank: NM_017460.6); HNF4A (forward primer: ACT​ACG​GTG​CCT​CGA​GCT​GT; reverse primer: GGC​ACT​GGT​TCC​TCT​TGT​CT; GenBank: NM_178849.3); albumin (ALB, forward primer: ATG​CTG​AGG​CAA​AGG​ATG​TC; reverse primer: AGC​AGC​AGC​ACG​ACA​GAG​TA; GenBank: NM_000477.7). Total RNA from HLiOs was extracted using the miRNeasy Mini Kit (QIAGEN, Germany) according to the manufacturer’s instructions. RNA purity and concentration were assessed using a NanoDrop spectrophotometer (Thermo Fisher Scientific, United States). Then, 200 ng of total RNA was reverse-transcribed into single-stranded cDNA using a High-Capacity RNA-to-cDNA Kit (Thermo Fisher Scientific, United States). Gene expression analysis was performed using a StepOnePlus Real-Time PCR System (Thermo Fisher Scientific, United States). GAPDH (forward primer: TCA​AGA​AGG​TGG​TGA​AGC​AGG; reverse primer: ACC​AGG​AAA​TGA​GCT​TGA​CAA​A; GenBank: NM_002046.6) was used as the reference gene. The relative mRNA expression levels were calculated using the 2^−ΔΔCt^ method.

### 2.5 Analysis of secreted proteins

The concentrations of high mobility group box 1 (HMGB1), interleukin one beta (IL-1β), interleukin 8 (IL-8) and lectin-like oxidized low-density lipoprotein receptor-1 (LOX-1) at each time-point (pre-ischemia, post-ischemia, IRI 6 h and IRI 24 h) were determined using a magnetic bead-based multiplex immunoassay from Luminex with Human ProcartaPlex (Thermo Fisher Scientific, United States) according to the manufacturer’s instructions. The concentration of each factor was calculated from the standard curves. Data acquisition was performed using a Luminex xMAP INTELLIFLEX System (Luminex Corporation, United States).

### 2.6 Analysis of albumin secretion

We examined the mature phenotype of the HLiOs by analyzing albumin production and secretion. Briefly, liver organoids were grown in EM or DM and the culture supernatant was collected 48 h after the last medium change. The amount of albumin was determined using the Human Albumin ELISA Kit (Abnova, Taiwan), according to the manufacturer’s instructions.

### 2.7 Immunofluorescence analysis

For immunofluorescence staining, HLiOs were recovered from BME, and fixed with 4% paraformaldehyde (Sigma-Aldrich, United States) for 45 min on ice. The samples were then permeabilized with 0.1% Triton X-100% and 1% BSA in DPBS (Thermo Fisher Scientific, United States) for 40 min at room temperature (RT). After permeabilization, the liver organoids were washed with DPBS and blocked for 1 h at RT in blocking solution containing 1% BSA in DPBS. HLiOs were subsequently incubated overnight at 4 °C with the following primary antibodies diluted in blocking solution: anti-human CD326 (EpCAM, 1:200, PA5-21004, Thermo Fisher Scientific); anti-human albumin (ALB, 1:200, GTX102419, GeneTex); anti-human cytochrome P450 3A4 (CYP3A4, 1:50, MA5-17064, Thermo Fisher Scientific); anti-human alpha-1 antitrypsin (A1AT, 1:200, EPR9090, Abcam); anti-human hepatocyte nuclear factor 4 alpha (HNF4α, 1:100, EPR3648, Abcam); and anti-human leucine-rich repeat-containing G-protein-coupled receptor 5 (LGR5, 1:200, MA5-25644, Thermo Fisher Scientific). After washing with cold DPBS, HLiOs were incubated for 1 h at RT with goat anti-rabbit or anti-mouse IgG secondary antibodies conjugated with Cy3 or fluorescein, respectively (AffiniPure goat anti-rabbit or anti-mouse IgG, Jackson ImmunoResearch, United States), at a concentration of 10 μg/mL, together with 4′,6-diamidino-2-phenylindole (DAPI; 1 μg/mL; Sigma-Aldrich, United States). Following an additional DPBS wash, the samples were mounted using Vectashield Antifade Mounting Medium (Vector Laboratories, United States). The samples were imaged using a Leica SP5 confocal system mounted on a Leica DM6000 inverted microscope (Leica Microsystems, United States). Fluorescence intensity was quantified using ImageJ software (v1.54, NIH, United States).

### 2.8 Cell viability assay

The viability of HLiOs was determined using the CellTiter-Glo 3D Cell Viability Assay according to the manufacturer’s instructions (Promega, United States). Briefly, the CellTiter-Glo lysis reagent was added to the organoid culture medium at a 1:1 ratio. The HLiOs were disrupted vigorously for 5 min, and then incubated at RT for 30 min in the dark. Luminescence was determined using a Spark Multimode Microplate Reader (Tecan, Switzerland).

### 2.9 Statistical analysis

All experiments were done at least three times independently. Data are presented as mean ± standard deviation (SD). Statistical analyses were conducted using GraphPad Prism software (version 10; GraphPad Software, United States). Comparisons among multiple groups were performed using one-way analysis of variance (ANOVA). When ANOVA indicated statistical significance, *post hoc* multiple comparison tests were applied as appropriate (Tukey’s or Dunnett’s test) to assess the differences between groups. Statistical significance was set at p < 0.05.

## 3 Results

### 3.1 Establishment of undifferentiated human liver organoids and induction of hepatocyte differentiation

**FIGURE 1:**
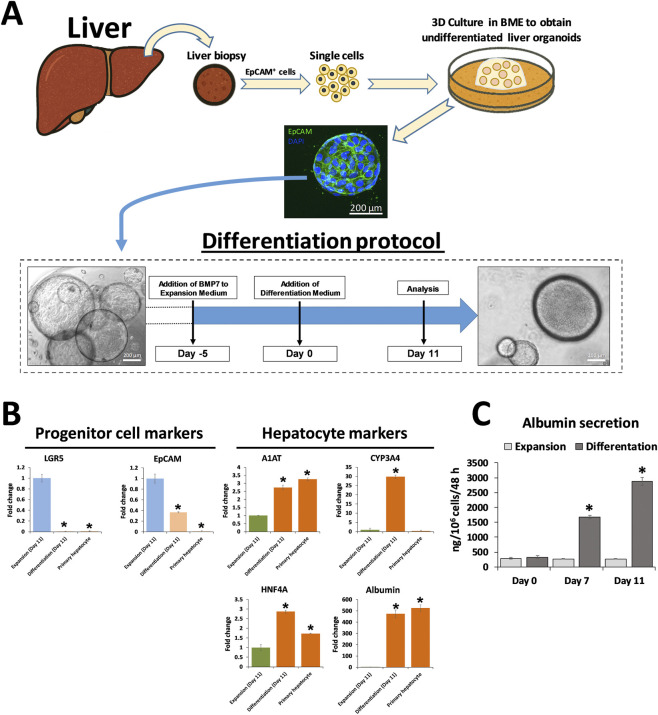
Generation of human liver organoids and induction of hepatocyte differentiation. **(A)** Schematic overview of the workflow used to establish human liver organoids (HLiOs) and the subsequent differentiation protocol. EpCAM^+^ cells isolated from human liver biopsies were cultured to generate undifferentiated 3D liver organoids that were enriched in progenitor-like cells. Representative immunofluorescence images showing EpCAM expression (green) and nuclei (DAPI, blue) confirm the progenitor phenotype of the expanding organoids (scale bar: 200 µm). Differentiation was induced by BMP7 supplementation during the expansion phase (day 5), followed by exposure to hepatocyte differentiation medium (day 0), with analyses performed on day 11. Representative bright-field images illustrate organoid morphology during expansion and after differentiation (scale bars: 200 µm). **(B)** Gene expression analysis of progenitor and hepatocyte markers during cell expansion and differentiation. Primary human hepatocytes were included as reference control where indicated. Data are presented as fold change relative to expansion conditions. **(C)** Functional assessment of organoid maturation by measuring albumin secretion. Data are shown as the mean ± SD from at least three independent experiments. *p < 0.05 was considered significant vs. expansion condition.

HLiOs were successfully generated from EpCAM^+^ cells isolated from human liver biopsies, and expanded as 3D cultures in BME under defined conditions (Figure 1A). During the expansion phase, unlike classical compact multicellular spheroids, HLiOs formed lumen-containing epithelial structures generated through proliferative self-organization of EpCAM^+^ progenitor cells (Figure 1A). To promote hepatocyte maturation, organoids were exposed to a stepwise differentiation protocol consisting of BMP7 preconditioning followed by culture in hepatocyte differentiation medium (Figure 1A). Representative images correspond to selected experimental stages and not to continuous monitoring of the same organoids. Molecular characterization revealed a marked transition from a progenitor-like phenotype to a mature hepatocyte phenotype. Expression of progenitor-associated markers was markedly reduced upon differentiation, with LGR5 and EpCAM levels decreasing by 500-fold and 2.77-fold relative to expansion conditions, respectively (Figure 1B). Conversely, hepatocyte-related genes were strongly induced after differentiation, including albumin (474-fold), CYP3A4 (30-fold), HNF4A (2.9-fold), and A1AT (2.8-fold) compared with expansion medium, reaching levels comparable to or approaching those observed in primary hepatocytes (Figure 1B). Functional maturation was further supported by a progressive increase in albumin secretion over time. While albumin levels remained low and stable in expansion conditions (Day 0–11: 265–287 ng/10^6^ cells/48 h), differentiated organoids exhibited a marked increase at day 7 (1677 ng/10^6^ cells/48 h), and a further increase by day 11 (2879 ng/10^6^ cells/48 h), corresponding to approximately 6-fold and 10-fold increases relative to baseline, respectively (Figure 1C).

### 3.2 Validation of HLiO differentiation by confocal immunofluorescence analysis of progenitor and hepatocyte markers

**FIGURE 2:**
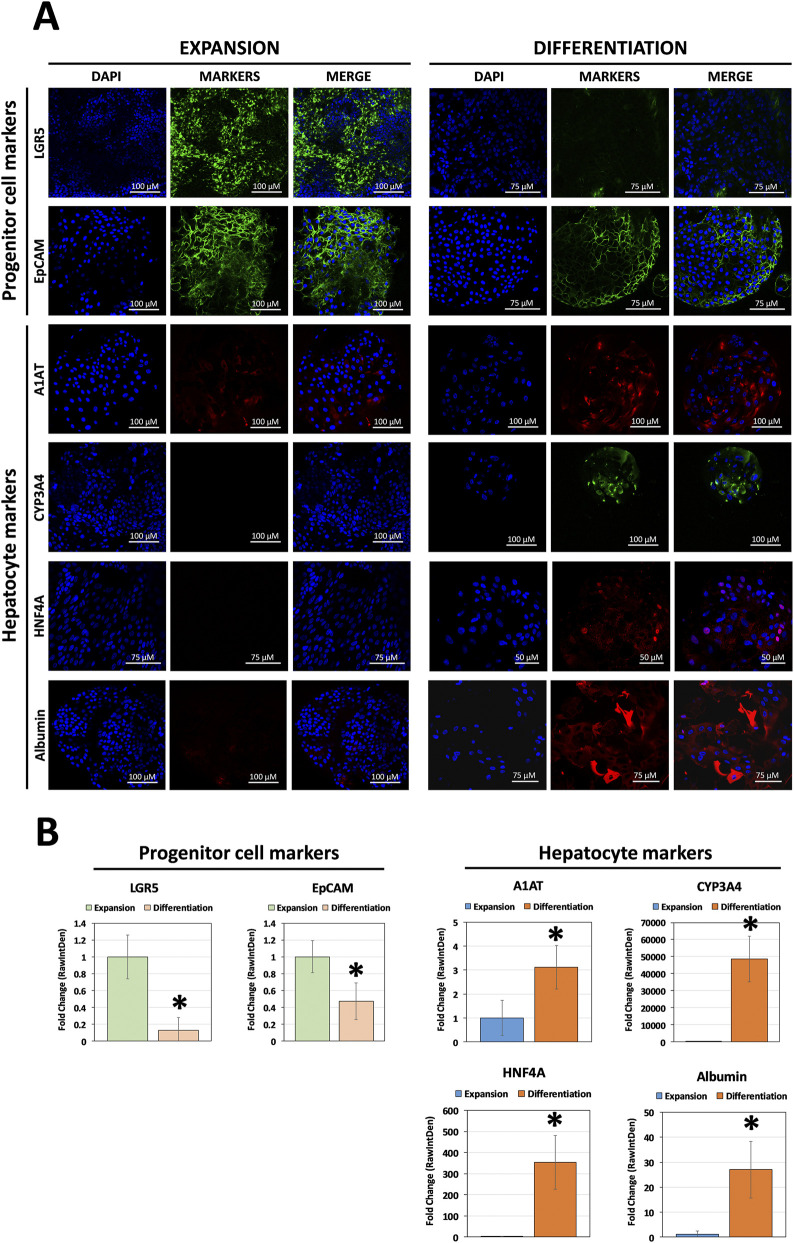
Validation of progenitor and hepatocyte marker expression in human liver organoids using confocal immunofluorescence analysis. **(A)** Representative confocal immunofluorescence images showing the expression of progenitor cell markers (LGR5 and EpCAM), and hepatocyte markers (A1AT, CYP3A4, HNF4A, and albumin) in human liver organoids (HLiOs) cultured under expansion or differentiation conditions. Nuclei were counterstained with DAPI (blue), while specific markers are shown in green or red. Scale bars: 50–100 μm, as indicated. **(B)** Quantification of fluorescence intensity (RawIntDen) of progenitor and hepatocyte markers under expansion and differentiation conditions. Data are presented as fold changes relative to the expansion conditions. Data are shown as mean ± SD from at least three independent experiments. *p < 0.05 was considered significant vs. expansion condition.

To validate the transcriptional changes observed at the protein level during organoid differentiation, confocal immunofluorescence analysis was performed to assess the expression of progenitor and hepatocyte markers in HLiOs cultured under expansion or differentiation conditions (Figure 2). Undifferentiated organoids maintained in expansion medium showed strong expression of progenitor-associated markers, including LGR5 and EpCAM, confirming the preservation of a progenitor-like phenotype (Figure 2A). In contrast, organoids subjected to the differentiation protocol exhibited a marked reduction in LGR5 and EpCAM staining intensity, with quantitative fluorescence analysis showing a decrease of approximately 8.33-fold and 2.13-fold relative to the expansion conditions, respectively (Figure 2B). Consistent with gene expression data, hepatocyte-related proteins were minimally detected in expanding organoids, but became prominently expressed following differentiation. Specifically, differentiated organoids displayed robust staining for A1AT, CYP3A4, HNF4A, and albumin, indicating acquisition of mature hepatocyte features (Figure 2A). Quantitative fluorescence intensity analysis further confirmed the strong induction of hepatocyte markers, including HNF4A (354-fold), CYP3A4 (48.63-fold), A1AT (3.1-fold), and albumin (27-fold), compared with expansion conditions (Figure 2B). These findings confirm that EpCAM-derived HLiOs can be maintained in an undifferentiated progenitor-enriched state and eventually drive toward a mature hepatocyte phenotype through a defined differentiation protocol, thereby establishing a robust platform to investigate the effects of ischemic stress on liver regeneration and functional maturation.

### 3.3 Establishment and validation of a human liver organoid-based ischemia–reperfusion injury (IRI) model

**FIGURE 3:**
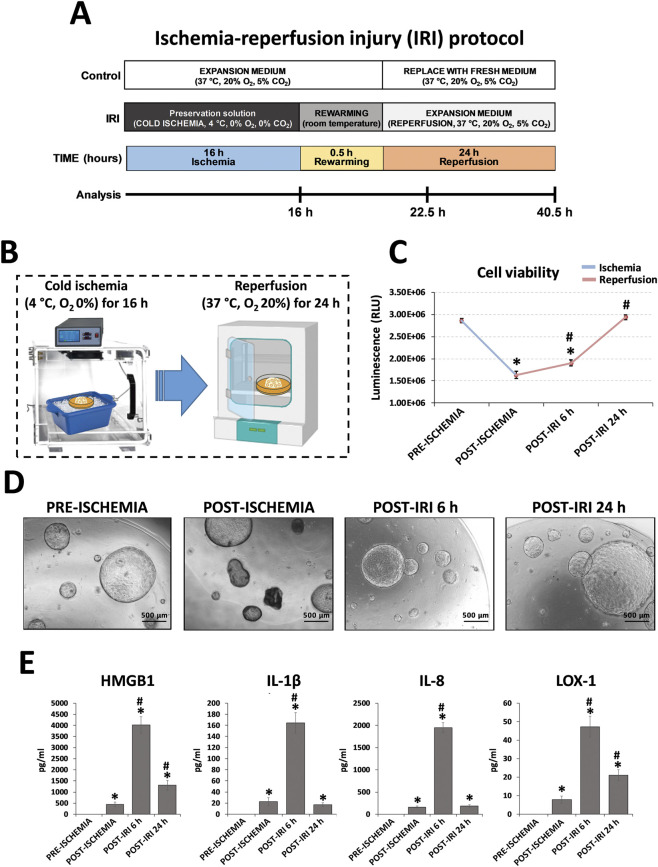
Establishment and validation of human liver organoid-based ischemia-reperfusion injury (IRI) model. **(A)** Schematic overview of the experimental workflow used to induce IRI in HLiOs. Control organoids were maintained in expansion medium under normoxic conditions (37 °C, 20% O_2_, 5% CO_2_), whereas the IRI group was subjected to 16-h cold ischemia in preservation solution (4 °C, 0% O_2_, 0% CO_2_), followed by 30 min of rewarming at room temperature, and subsequent reperfusion in expansion medium under normoxic conditions for up to 24 h. The time points for downstream analyses are indicated. **(B)** Graphical representation of the *in vitro* IRI procedure, illustrating the transition from cold ischemia to reperfusion conditions. **(C)** Cell viability analysis measured using luminescence-based assay (RLU). **(D)** Representative bright-field images of HLiOs before ischemia, immediately after ischemia, and during reperfusion (6 h and 24 h). Scale bars: 500 µm. **(E)** Quantification of key ischemia-reperfusion associated mediators (HMGB1, IL-1β, IL-8, and LOX-1) released by the HLiOs. Data are presented as the mean ± SD from at least three independent experiments. *p < 0.05 was considered significant vs. pre ischemia. ^#^p < 0.05 was considered significant vs. post ischemia. The incubator icon was obtained from BioRender.com and used under a valid BioRender license held by Dr. Giovanni Zito (2026).

To investigate the impact of ischemic stress on liver regeneration in a controlled *ex vivo* setting, we created a HLiO-based IRI model consisting of 16 h cold ischemia (4 °C, O_2_ 0%) followed by rewarming and 24 h of reperfusion (37 °C, O_2_ 20%) (Figures 3A,B). Preliminary optimization experiments (data not shown) tested multiple ischemic periods to achieve a sub-lethal injury compatible with subsequent recovery. Shorter ischemic intervals failed to induce significant damage, whereas prolonged ischemia resulted in irreversible organoid deterioration. A 16-h ischemic insult was therefore selected as an optimal condition capable of inducing substantial but reparable injury. Cell viability analysis revealed an approximately 45%–50% reduction immediately after ischemia, with luminescence values decreasing from 2.95 × 10^6^ RLU in pre-ischemic conditions to 1.63 × 10^6^ RLU post-ischemia (Figure 3C). During reperfusion, organoids progressively recovered viability, reaching 1.95 × 10^6^ RLU at 6 h, and nearly baseline levels by 24 h (2.93 × 10^6^ RLU), thus indicating efficient functional recovery. Morphological assessment by bright-field microscopy supported these findings, showing structural disruption and darkened organoid appearance after ischemia, followed by restoration of spherical morphology and structural integrity during reperfusion (Figure 3D). To further validate the IRI model, we quantified the release of key mediators associated with ischemia-reperfusion processes. The levels of HMGB1, IL-1β, IL-8, and LOX-1 were markedly induced following ischemic insult and peaked predominantly at early reperfusion (IRI 6 h), with HMGB1 reaching 4023 pg/mL, IL-1β 164 pg/mL, IL-8 1953 pg/mL, and LOX-1 47 pg/mL, respectively (Figure 3E). Notably, mediator levels declined substantially after 24 h of reperfusion (HMGB1: 1306 pg/mL; IL-1β: 17 pg/mL; IL-8: 190 pg/mL; LOX-1: 21 pg/mL), consistent with the resolution of acute stress and recovery of organoid homeostasis. These results demonstrate the successful establishment of an *ex vivo* IRI model using an HLiO-based platform that recapitulates the key hallmarks of clinically relevant IRI, including transient loss of viability, morphological damage, and induction of inflammatory and damage-associated mediators, while preserving the capacity of recovery upon reperfusion.

### 3.4 Cold ischemia enhances hepatocyte differentiation of human liver organoids

**FIGURE 4:**
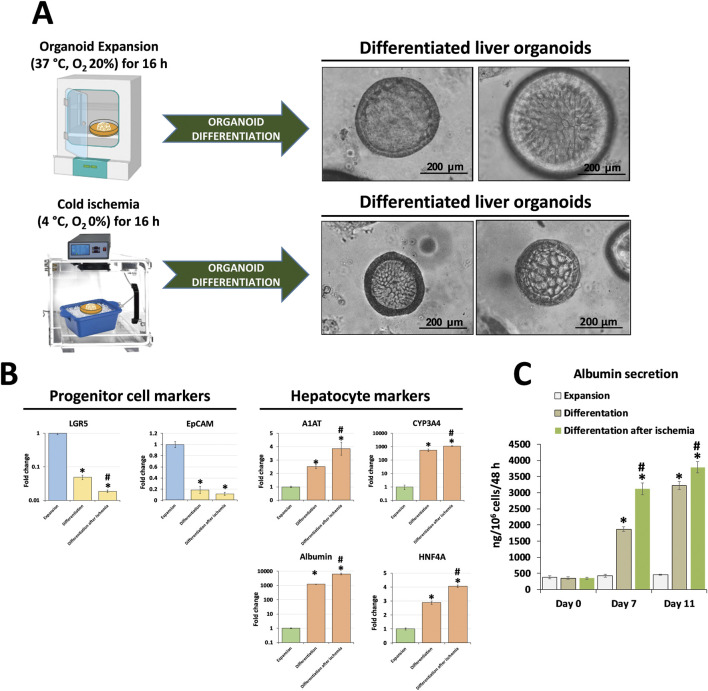
Cold ischemia enhances hepatocyte differentiation and functional maturation of human liver organoids. **(A)** Experimental design illustrating the impact of cold ischemia on the differentiation capacity of HLiOs. Undifferentiated organoids were either maintained under standard expansion conditions (37 °C, 20% O_2_) or subjected to 16 h of cold ischemia (4 °C, 0% O_2_) prior to the induction of hepatocyte differentiation. Representative bright-field images show organoid morphology after differentiation. Scale bars: 200 µm. **(B)** Gene expression analysis of progenitor and hepatocyte markers during expansion, differentiation, and differentiation following ischemia. Data are presented as fold changes relative to the expansion conditions. **(C)** Functional assessment of hepatocyte maturation by measuring albumin secretion. Data are shown as the mean ± SD from at least three independent experiments. *p < 0.05 vs. expansion; ^#^p < 0.05 vs. differentiation. The incubator icon was obtained from BioRender.com and used under a valid BioRender license held by Dr. Giovanni Zito (2026).

To investigate whether ischemic stress influenced the regenerative differentiation capacity of HLiOs, undifferentiated organoids were subjected to 16 h of cold ischemia before differentiation induction (Figure 4A). Morphological assessment by bright-field microscopy (performed at the end of the differentiation process) revealed that differentiated organoids previously exposed to ischemia displayed a more compact and structurally organized phenotype compared with differentiated organoids without ischemia, used as controls, suggesting enhanced acquisition of mature hepatocyte features (Figure 4A). Consistent with these observations, transcriptional analysis found a more pronounced reduction of progenitor-associated genes in organoids differentiated after ischemia, with LGR5 expression decreasing 55-fold compared with expansion conditions, versus 20-fold in standard differentiation (Figure 4B). In contrast, EpCAM expression was similarly reduced in both differentiated groups, and showed no significant differences between organoids differentiated after ischemia and non-ischemic differentiated organoids. Conversely, hepatocyte-related markers significantly increased following differentiation, and this effect was further amplified in organoids preconditioned by ischemia. In particular, CYP3A4 expression increased from 544-fold in standard differentiation to 1,108-fold after ischemia, while ALB expression ranged from 1,226-fold to 6,483-fold relative to expansion conditions. Similarly, HNF4A and A1AT showed enhanced induction after ischemic preconditioning (4-fold and 3.85-fold, respectively) compared to differentiation alone (2.89-fold and 2.51-fold, respectively). Functional maturation was further supported by increased albumin production and secretion in organoids differentiated after ischemia compared to that in non-ischemic differentiated organoids (Figure 4C). Notably, preliminary optimization experiments (data not shown) indicated that shorter ischemic periods did not significantly influence the differentiation potential of HLiOs, whereas longer ischemic periods resulted in excessive cell death and impaired organoid recovery, preventing subsequent differentiation assays.

### 3.5 Ischemic preconditioning enhances hepatocyte protein expression in differentiated human liver organoids

**FIGURE 5:**
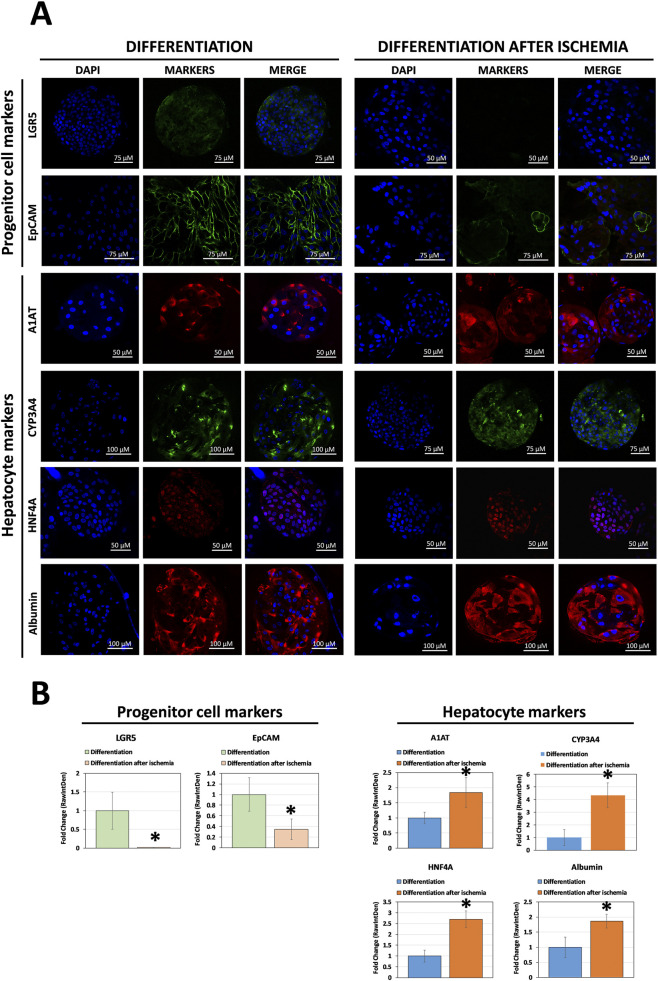
Validation of enhanced hepatocyte differentiation following ischemic preconditioning in human liver organoids. **(A)** Representative confocal immunofluorescence images showing the expression of progenitor cell markers (LGR5 and EpCAM) and hepatocyte markers (A1AT, CYP3A4, HNF4A, Albumin) in HLiOs differentiated under standard conditions or 16 h of cold ischemia prior to differentiation. Nuclei were counterstained with DAPI (blue), and specific markers are shown in green or red. Scale bars: 50–100 μm, as indicated. **(B)** Quantification of fluorescence intensity (RawIntDen) of progenitor and hepatocyte markers in differentiated organoids with or without ischemic preconditioning. Data are presented as fold change normalized to differentiation without ischemia. Data are shown as the mean ± SD from at least three independent experiments. *p < 0.05 was considered statistically significant vs. differentiation without ischemic preconditioning.

To validate the transcriptional changes induced by ischemic preconditioning before differentiation at the protein level, confocal immunofluorescence analysis was performed to assess progenitor and hepatocyte marker expression in HLiOs differentiated with or without prior exposure to cold ischemia (Figure 5). Organoids differentiated under standard conditions exhibited a minimal abundance of the progenitor marker LGR5 and low EpCAM levels, together with clearly detectable staining for hepatocyte markers (A1AT, CYP3A4, HNF4A, and albumin), consistent with the acquisition of a mature phenotype (Figure 5A). Notably, organoids differentiated after ischemia exhibited a further decrease in progenitor-associated proteins, and quantitative fluorescence analysis showed a significant reduction in the LGR5 signal (62.5-fold relative to differentiation alone) and a moderate decrease in EpCAM (2.9-fold), supporting enhanced loss of the progenitor-like state. In parallel, hepatocyte-associated proteins significantly increased following ischemic preconditioning. Differentiation after ischemia resulted in a higher fluorescence intensity of HNF4A (2.70-fold), CYP3A4 (4.32-fold), A1AT (1.84-fold), and albumin (1.86-fold) compared to non-ischemic differentiated organoids (Figure 5B).

## 4 Discussion

In solid organ transplantation (SOT), IRI develops upon restoration of vascular perfusion after graft implantation and reflects the cumulative effects of ischemic insults occurring throughout the transplant process. These include cold ischemia during graft preservation and transportation, as well as warm ischemia during procurement and implantation, which together contribute to metabolic stress and subsequent reperfusion-associated injury (Gilbo et al., 2023; Chullo et al., 2024). Although SOT donor and recipient characteristics influence susceptibility to injury (Fernandez et al., 2020; Miceli et al., 2025), ischemic duration remains a key determinant of graft damage (Jernryd et al., 2020; Cesaretti et al., 2024). During procurement, grafts undergo static cold storage, an inexpensive and widely used strategy to limit IRI. However, it preserves organs only for a limited time, as cold ischemic injury progressively accumulates (Abbas et al., 2024; Chullo et al., 2024). In this context, while minimizing ischemic time is widely recognized as critical for optimal outcomes, it remains unclear whether defined temporal ischemic windows simultaneously limit detrimental effects while activating adaptive regenerative mechanisms that enhance graft recovery.

Liver regeneration plays a central role in the restoration of hepatic function after resection or transplantation (Fausto et al., 2006; Haga et al., 2008; Michalopoulos and Bhushan, 2021; Vosough et al., 2025). Importantly, the pathways governing regeneration overlap with those involved in responses to ischemic stress (Konishi and Lentsch, 2017; Pretzsch et al., 2022), suggesting that IRI and regenerative signaling are mechanistically interconnected. In this regard, controlled injury may trigger the release of regenerative cytokines and growth factors, enhance adaptive stress responses, and promote the reactivation of hepatocyte cell-cycle and progenitor differentiation (Konishi and Lentsch, 2017; Pretzsch et al., 2022; Zhang et al., 2024a; Zhang et al., 2024b). These observations support the concept that ischemic stress is a double-edged sword; excessive injury is deleterious, whereas appropriately modulated stress may positively influence regenerative outcomes.

To explore this balance, we developed a HLiO-based IRI model to investigate the impact of cold ischemia on liver regeneration. First, we established a HLiO model based on EpCAM^+^ cells (Figure 1), which are widely recognized as liver progenitor cells capable of differentiating into cholangiocytes and hepatocytes (Segal et al., 2019; Safarikia et al., 2020). HLiOs can be generated from multiple cell sources, including adult tissue-derived progenitors and iPSCs (Lo Nigro et al., 2021; Afonso et al., 2024). Although iPSC-derived systems provide remarkable developmental plasticity, maintaining a stable expandable undifferentiated hepatic progenitor state remains challenging (De Souza, 2017; Hofer and Lutolf, 2021). In our study, adult EpCAM^+^ progenitor-derived organoids provided a robust and reproducible platform for investigating regeneration-associated differentiation responses. This approach enabled the generation of undifferentiated organoids with preserved differentiation potential, thereby providing an *ex vivo* platform that recapitulates the key aspects of hepatic regeneration (Figure 2). To investigate the impact of ischemic stress on regenerative programs, we implemented an IRI protocol using this organoid system (Figure 3). To the best of our knowledge, this represents the first *ex vivo* regenerative liver organoid model that integrates a controlled ischemia-reperfusion paradigm to study the interplay between injury and differentiation. Our findings indicate that sub-lethal ischemia induces a transient injury response characterized by reduced viability and activation of inflammatory mediators, followed by rapid recovery during reperfusion (Figure 3). Notably, ischemic preconditioning enhanced hepatocyte maturation, as demonstrated by the strong downregulation of progenitor-associated programs, increased expression of hepatocyte-specific genes and proteins, and augmented albumin secretion (Figures 4, 5).

**FIGURE 6:**
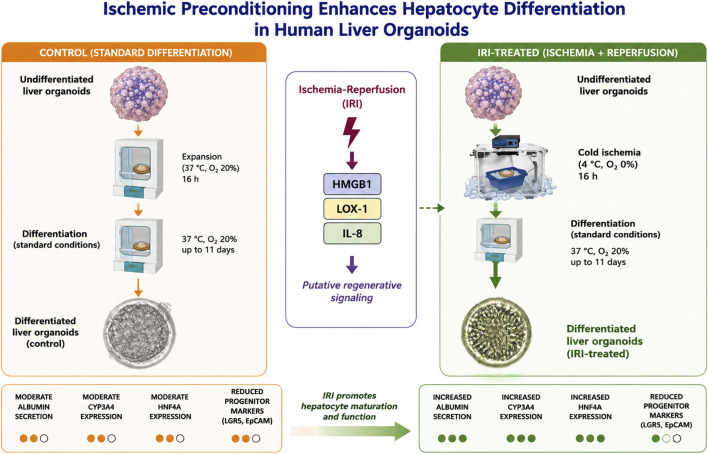
Schematic representation of the proposed mechanism by which controlled ischemia-reperfusion injury (IRI) may promote hepatocyte differentiation in human liver organoids (HLiOs). Undifferentiated liver progenitor organoids exposed to sub-lethal cold ischemia activate a stress-response signaling cascade characterized by the release of damage-associated molecular and inflammatory mediators, including HMGB1, LOX-1, and IL-8. This transient inflammatory and oxidative signaling environment may act as a regenerative priming stimulus that promotes the transition from a progenitor-like state toward hepatocyte maturation during liver regenerative responses. The incubator icon was obtained from BioRender.com and used under a valid BioRender license held by Dr. Giovanni Zito (2026).

Liver regenerative signaling following the first few hours of IRI is orchestrated by a complex interplay between cytokines, growth factors, and metabolic cues (Fausto et al., 2006; Pretzsch et al., 2022). Classical mediators, such as TNF-α and IL-6, are known to prime hepatocytes for proliferation through NF-kB and STAT3 signaling pathways (Fausto et al., 2006; Pretzsch et al., 2022; Vosough et al., 2025). In particular, TNF-α binds its receptor on non-parenchymal liver Kupffer cells, and stimulates the production of IL-6 via NF-kB activation. IL-6 acts directly on hepatocytes and induces a cascade of events that lead to the progression of the cell cycle. However, our organoid system represents a simplified regenerative niche dominated by EpCAM^+^ liver progenitor cells (Segal et al., 2019; Safarikia et al., 2020), and lacks the full spectrum of non-parenchymal cell populations. Consistent with this cellular composition, our model did not exhibit detectable TNF-α or IL-6 production (data not shown), but instead displayed robust production of HMGB1, IL-1β, IL-8, and LOX-1. This mediator’s profile may well represent a progenitor-driven inflammatory program distinct from the canonical hepatocyte and Kupffer cell-mediated responses observed *in vivo*. In particular, HMGB1, LOX-1, and IL-8 likely constitute a coordinated signaling axis linking oxidative stress, inflammation, and differentiation. HMGB1 has been increasingly implicated in liver progenitor cell-associated regenerative responses, and transient HMGB1 signaling may promote ductular activation, progenitor plasticity, and differentiation competence through RAGE- and TLR4-dependent pathways (Hernandez et al., 2018). Released rapidly upon cellular stress, HMGB1 acts as a DAMP that activates NF-kB, promoting inflammatory and regenerative priming responses mediated by Kupffer cells and liver progenitor cells, respectively (Tsung et al., 2005; Hernandez et al., 2018). Although LOX-1 has been studied primarily in endothelial and inflammatory contexts (Sanchez-Leon et al., 2024), emerging evidence suggests that LOX-1 can also be expressed by hepatocytes (Wang et al., 2022), and oxidative stress-induced LOX-1 signaling may influence regenerative processes in the liver (Huang et al., 2025). In ischemic injury, LOX-1 may act as a redox-sensitive amplifier of NF-kB–dependent pathways (Cominacini et al., 2000), potentially contributing to the establishment of an inflammatory niche that supports regenerative activation and differentiation (Kitade et al., 2016). Within this stress-responsive microenvironment, IL-6 is the classical cytokine involved in hepatocyte priming and proliferation during liver regeneration, predominantly produced by Kupffer cells and mature hepatocytes (Camargo et al., 1997; Fausto et al., 2006; Pretzsch et al., 2022; Vosough et al., 2025). In our model, we did not observe IL-6 production, probably because of the lack of mature hepatocytes and Kupffer cells. In contrast, IL-8 emerged as a key progenitor-associated effector cytokine. IL-8 is primarily produced by hepatic stellate cells, and the CXCL8–CXCR1/CXCR2 signaling axis contributes to neutrophil recruitment and inflammatory orchestration during liver regeneration (Yan et al., 2025). IL-8 is markedly upregulated in acute liver failure and severe acute liver injury (Sasaki et al., 2019), and has been shown to promote phenotypic plasticity by inducing the transdifferentiation of mature hepatocytes toward a cholangiocyte-like lineage (Sasaki et al., 2019). Consistent with this, emerging evidence indicates that epithelial progenitor-like liver cells participate in inflammatory cytokine networks, with IL-8 associated with progenitor-like phenotypic transitions, epithelial inflammatory signaling, and ductular reactions during liver injury (Sasaki et al., 2019; Huang et al., 2023). Collectively, these findings suggest that beyond its classical inflammatory role, IL-8 may support progenitor survival, migration, and differentiation competence (Sasaki et al., 2019; Yan et al., 2025), raising the possibility that during ischemic stress transient IL-8 serves as a regenerative priming signal rather than solely as a pro-inflammatory mediator. The presence of IL-8, but not IL-6, in our model aligns with a progenitor-driven regenerative program, instead of a mature hepatocyte-dependent regenerative response. Mechanistically, this HMGB1–LOX-1–IL-8 axis may operate as an intrinsic stress-response module within liver progenitor cells, coupling oxidative damage sensing with the activation of pro-regenerative signaling pathways. Beyond a purely inflammatory cascade, the coordinated production of these mediators by progenitor cells may create a permissive microenvironment that favors transition from a progenitor-like state toward hepatocyte maturation. Taken together, our data support a model in which HMGB1 and LOX-1 act as upstream stress sensors, while IL-8 functions as a downstream effector the shapes progenitor behavior. Controlled ischemic stress appears to modulate the temporal dynamics of this axis, and early activation of HMGB1–LOX-1–IL-8 signaling may prime regenerative pathways, whereas subsequent attenuation during reperfusion permits progression toward terminal hepatocyte maturation. A schematic representation of the proposed regenerative signaling model is shown in Figure 6. In this context, ischemic preconditioning enhances differentiation efficiency and reduces progenitor marker expression, suggesting that regulated stress exposure can coordinate the transition from progenitor activation to functional maturation.

Despite providing novel insights into ischemia-driven regenerative priming in HLiOs, this study has several limitations that should be considered. First, although organoid viability was quantitatively assessed using a whole-structure ATP-based assay, complementary spatial live/dead approaches (e.g., Calcein-AM/Ethidium staining) may provide additional information regarding regional viability patterns within organoid structures. Second, our organoid system represents a simplified model predominantly composed of EpCAM^+^ liver progenitor-derived cells and therefore does not fully recapitulate the cellular complexity of the native liver microenvironment. Furthermore, the relatively short differentiation period adopted in this study resulted in predominantly spherical organoid structures with limited higher-order architectural complexity. Although this approach was intentionally selected to preserve differentiation stability and viability during the experimental timeline, longer maturation periods may promote further structural complexity and tissue organization. Integration of more advanced multicellular systems, as well as perfused microfluidic or organ-on-chip platforms, may further improve physiological relevance and enable investigation of additional IRI-associated factors such as flow-mediated shear stress and multicellular interactions. In addition, the use of continuous live time-lapse imaging approaches could provide dynamic information regarding organoid behavior and differentiation trajectories during ischemia and recovery phases. Third, although our data support a potential role for the HMGB1–LOX-1–IL-8 axis in promoting hepatocyte maturation, the study remains descriptive and does not include pathway inhibition or loss-of-function experiments required to establish direct causal relationships. Finally, future implementation of advanced omics approaches, including single-cell RNA sequencing and multi-omics profiling, may provide high-resolution characterization of cellular heterogeneity, differentiation trajectories, and molecular mechanisms underlying ischemia-induced regenerative responses.

## 5 Conclusion

The *ex vivo* IRI platform developed here provides a simplified yet physiologically relevant system to dissect regenerative mechanisms triggered by ischemia. Although it does not fully recapitulate the multicellular complexity of the liver, it allows the identification of progenitor-specific signaling pathways that may otherwise be masked *in vivo*. From a translational perspective, our results show that controlled ischemic cues or ischemia-mimetic strategies might be managed to enhance graft regeneration and functional reprogramming, although further studies are needed to dissect the molecular pathways underlying this priming effect, and to determine their relevance *in vivo*.

## Data Availability

The original contributions presented in the study are included in the article/supplementary material, further inquiries can be directed to the corresponding author.

## Glossary

IRI: ischemia-reperfusion injury
HLiO: human liver organoid
LTx: liver transplantation
DAMPs: damage-associated molecular patterns
EBSS: Earle’s balanced salt solution
PBS: phosphate-buffered saline
EpCAM: epithelial cell adhesion molecule
FACS: fluorescence-activated cell sorting
BME: basement membrane extract
EM: expansion medium
DM: differentiation medium
HEPES: 4-(2-hydroxyethyl)-1-piperazineethanesulfonic acid
EGF: epidermal growth factor
FGF: fibroblast growth factor
HGF: hepatocyte growth factor
RSPO1: R-spondin-1
BMP: bone morphogenetic protein
BSA: bovine serum albumin
ALB: albumin
CYP3A4: cytochrome P450 3A4
A1AT: alpha-1 antitrypsin
HNF4α: hepatocyte nuclear factor 4 alpha
LGR5: leucine-rich repeat-containing G-protein-coupled receptor 5
DAPI: 4′:6-diamidino-2-phenylindole
RT-qPCR: Real-time quantitative PCR
GAPDH: Glyceraldehyde 3-phosphate dehydrogenase
HMGB1: high mobility group box 1
IL-1β: interleukin 1 beta
IL-8: interleukin 8
LOX-1: lectin-like oxidized low-density lipoprotein receptor-1
SD: standard deviation
ANOVA: analysis of variance
RLU: relative light units
SOT: solid organ transplantation
DCD: donation after circulatory death
DBD: donation after brain death
